# Nomograms to estimate long-term overall survival and breast cancer-specific survival of patients with luminal breast cancer

**DOI:** 10.18632/oncotarget.7975

**Published:** 2016-03-07

**Authors:** Wei Sun, Yi-Zhou Jiang, Yi-Rong Liu, Ding Ma, Zhi-Ming Shao

**Affiliations:** ^1^ Department of Breast Surgery, Fudan University Shanghai Cancer Center, Shanghai, P.R. China; ^2^ Cancer Institute, Fudan University Shanghai Cancer Center, Shanghai, P.R. China; ^3^ Department of Oncology, Shanghai Medical College, Fudan University, Shanghai, P.R. China; ^4^ Institutes of Biomedical Sciences, Fudan University, Shanghai, P.R. China

**Keywords:** nomogram, luminal breast cancer, overall survival, breast cancer-specific survival

## Abstract

Luminal breast cancer constitutes a group of highly heterogeneous diseases with a sustained high risk of late recurrence. We aimed to develop comprehensive and practical nomograms to better estimate the long-term survival of luminal breast cancer.

Patients with luminal breast cancer diagnosed between 1990 and 2006 were retrieved from the Surveillance, Epidemiology, and End Results (SEER) database and randomly divided into the training (*n* = 87,867) and validation (*n* = 88,215) cohorts. The cumulative incidence function (CIF) and a competing-risks model were used to estimate the probability of breast cancer-specific survival (BCSS) and death from other causes. We integrated significant prognostic factors to build nomograms and subjected the nomograms to bootstrap internal validation and to external validation.

We screened 176,082 luminal breast cancer cases. The 5- and 10-year probabilities of overall death were 0.089 and 0.202, respectively. The 5- and 10-year probabilities of breast cancer-specific mortality (BCSM) were 0.053 and 0.112, respectively. Nine independent prognostic factors for both OS and BCSS were integrated to construct the nomograms. The calibration curves for the probabilities of 5- and 10-year OS and BCSS showed excellent agreement between the nomogram prediction and actual observation. The C-indexes of the nomograms were high in both internal validation (0.732 for OS and 0.800 for BCSS) and external validation (0.731 for OS and 0.794 for BCSS).

We established nomograms that accurately predict OS and BCSS for patients with luminal breast cancer. The nomograms can identify patients with higher risk of late overall mortality and BCSM, helping physicians in facilitating individualized treatment.

## INTRODUCTION

Breast cancer is the most common cancer diagnosed in women, [1] and approximately 1 in 8 women living in the United States has a lifetime risk of being diagnosed with breast cancer. [2] It has been well established that these tumors are extremely heterogeneous in that their gene-expression profiles vary between individuals. [3, 4] One type of breast cancer, hormone receptor (HoR) positive, is known as luminal breast cancer and represents approximately two-thirds of all cases. [5] When comparing with triple-negative and human epidermal growth factor receptor 2 positive breast cancers, luminal breast cancer has more therapeutic options, including hormonal therapy and even targeted therapy. However, after receiving 5 years of adjuvant hormonal therapy, patients with luminal breast cancer still have a sustained risk of disease recurrence and death for at least 15 years after diagnosis. [6] Both the Arimidex, Tamoxifen, Alone or in Combination (ATAC) trial and the Breast International Group (BIG) 1-98 study have shown a continuing rate of recurrence, and more than half of all relapse events occurred in luminal breast cancer patients even 5 years after initial treatment. [7–9] Luminal breast cancer consists of a group of highly heterogeneous diseases, and the prognosis of each patient is extremely variable. [10] Therefore, it is of great importance to screen out patients with a high risk of late recurrence and a poor prognosis so more aggressive treatments can be applied.

Given the potential for a relatively long-term survival of patients with luminal breast cancer, a considerable number of patients might die from other causes. As a result, overall survival (OS) might fail to accurately describe a patient's long-term survival rate attributed to breast cancer. Thus, taking other causes of death into consideration is necessary when estimating breast cancer-specific survival (BCSS).

Nomograms have been widely used in clinical oncology as reliable and convenient tools for quantifying risk by incorporating and illustrating important prognostic factors. However, to the best of our knowledge, nomograms for predicting long-term OS and BCSS of patients with luminal breast cancer have not been reported. In this study, we aimed to develop comprehensive and practical nomograms based on a large population with long-term follow-up to better estimate the long-term OS and BCSS of luminal breast cancer patients.

## RESULTS

### Patient characteristics

**Figure 1:**
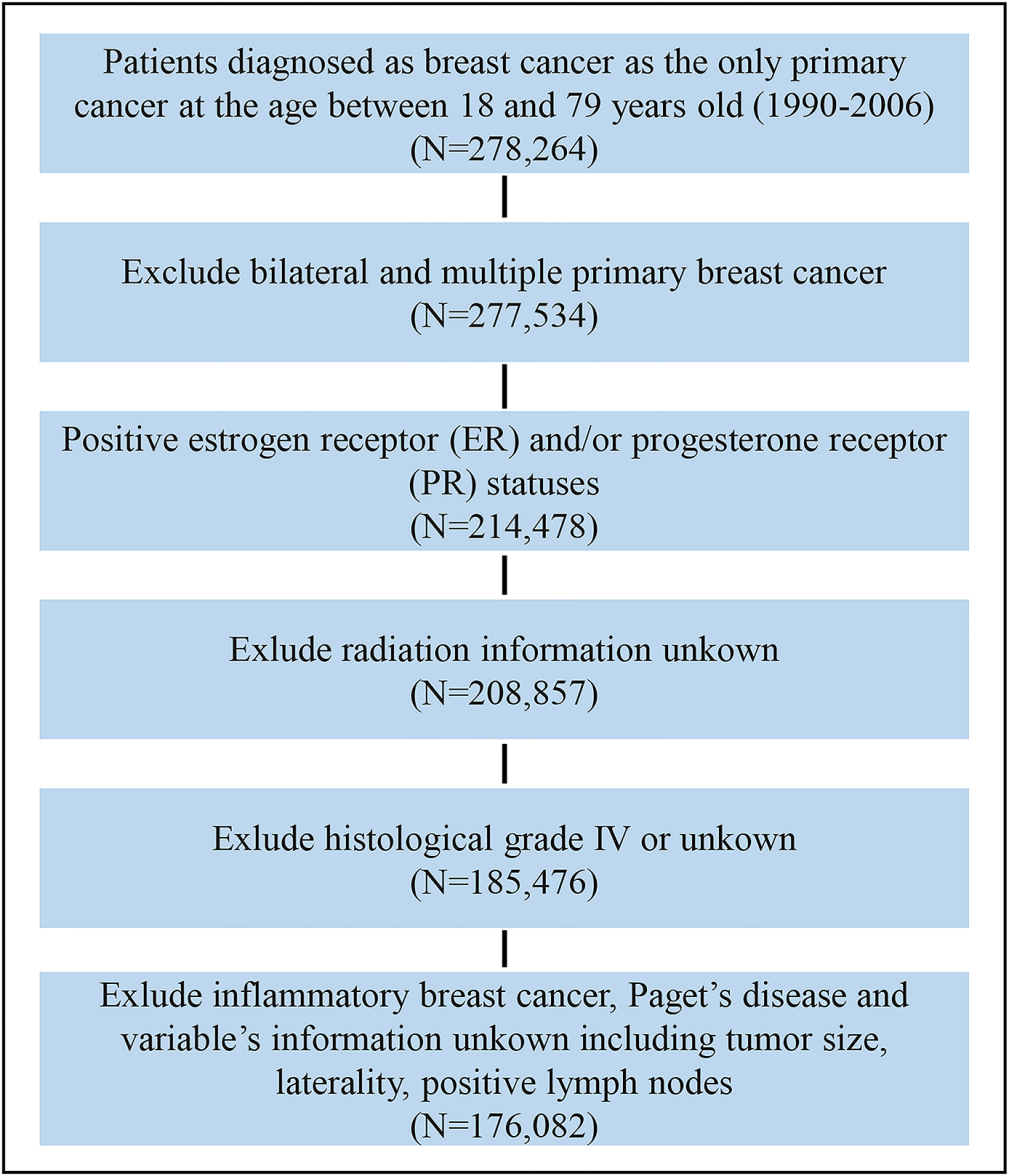
Flow chart for the SEER data screening

The entire population were collected from the Surveillance, Epidemiology, and End Results (SEER) program, containing 176,082 patients with histologically confirmed invasive breast cancer, with 87,867 patients in the training cohort and 88,215 patients in the validation cohort. The flow chart for SEER data selection is shown in Figure 1. The clinicopathological characteristics of these patients are listed in Table 1. The median ages at diagnosis of the training and validation cohorts were 57 years (25%–75%, 48–67 years), respectively. The median survival times of the training and validation cohorts were 100 months (25%–75%, 73–134 months), respectively. By the end of the last follow-up, 36,911 (21.0%) patients of the entire population had died, including 17,855 (10.1%) from breast cancer and 19,056 (10.8%) from other causes.

**Table 1:** Patients’ demographics and clinical characteristics

Variable	All Patients (*n* = 176,082)		Training Cohort (*n* = 87,867)		Validation Cohort (*n* = 88,215)	
	No.	%	No.	%	No.	%
Age at diagnosis, years						
Median (IQR)		57 (48–67)		57 (48–67)		57 (48–67)	
**Race**						
White	149,284	0.85	74,468	0.85	74,816	0.85
Black	12,155	0.07	6,082	0.07	6,073	0.07
Other*	14,643	0.08	7,317	0.08	7,326	0.08
**Laterality**						
Left	89,085	0.51	44,477	0.51	44,608	0.51
Right	86,997	0.49	43,390	0.49	43,607	0.49
**Tumor Size (cm)**						
≤ 2	118,606	0.67	59,118	0.67	59,488	0.67
2–5	48,528	0.28	24,190	0.28	24,338	0.28
> 5	8,948	0.05	4,559	0.05	4,389	0.05
**Histology**						
IDC	131,173	0.74	65,604	0.75	65,569	0.74
ILC	11,612	0.07	5,803	0.07	5,809	0.07
Mix/Other	33,297	0.19	16,460	0.19	16,837	0.19
**Grade**						
I	41,432	0.24	20,662	0.24	20,770	0.24
II	85,559	0.49	42,690	0.49	42,869	0.49
III	49,091	0.28	24,515	0.28	24,576	0.28
**Positive Lymph nodes**						
0	114,387	0.65	57,092	0.65	57,295	0.65
1–3	41,847	0.24	20,710	0.24	21,137	0.24
> 3	19,848	0.11	10,065	0.11	9,783	0.11
**ER Status**						
Positive	171,216	0.97	85,450	0.97	85,766	0.97
Negative	4,866	0.03	2,417	0.03	2,449	0.03
**PR Status**						
Positive	149,054	0.85	74,342	0.85	74,712	0.85
Negative	27,028	0.15	13,525	0.15	13,503	0.15
**Radiation**						
Yes	101,480	0.58	50,791	0.58	50,689	0.57
No	74,602	0.42	37,076	0.42	37,526	0.43
**Survival Months**						
Median (IQR)	100 (73–134)		100 (73–134)		100 (73–134)	

* Other including American Indian/AK Native, Asian/Pacific Islander. Abbreviations: ER, estrogen receptor; IDC, infiltrating ductal carcinoma; ILC, infiltrating lobular carcinoma; PR, progesterone receptor; IQR, interquartile range.

### Factors associated with OS

For the training cohort, data on the age at diagnosis, race, laterality of breast cancer, tumor size, histology type, tumor grade, number of positive lymph nodes, ER status, PR status and treatment with radiation therapy were collected. These variables, except laterality of breast cancer, proved to be significantly correlated with OS in univariate survival analysis (*P*'s < 0.001 for all except histology type and *P* = 0.002 for histology type). These prognostic factors that were identified in univariate analysis were included in the multivariate analysis (Cox proportional hazards model) and were further confirmed to be independently associated with OS (Table 2). These factors were then included in the nomogram. A weighted total score calculated from these variables was used to estimate 5- and 10-year OS.

**Table 2:** Univariate and multivariate analyses of overall survival in the training cohort

Variable	Univariate Analysis	Multivariate Analysis	
	*P* value	HR (95% CI)	*P* value
Age at diagnosis, years	< 0.001		
< 40		1.477 (1.388–1.571)	< 0.001
40–49		Reference	
50–59		1.160 (1.102–1.222)	< 0.001
60–69		2.037 (1.939–2.141)	< 0.001
70–79		4.619 (4.412–4.836)	< 0.001
Race	< 0.001		
White		Reference	
Black		1.492 (1.420–1.567)	< 0.001
Other		0.850 (0.802–0.901)	< 0.001
Laterality	0.612		
Left			
Right			
Tumor Size (cm)	< 0.001		
≤ 2		Reference	
2–5		1.509 (1.460–1.561)	< 0.001
> 5		2.132 (2.017–2.253)	< 0.001
Histology type	0.002		
IDC		Reference	
ILC		0.868 (0.818–0.921)	< 0.001
Mix/Other		0.943 (0.907–0.981)	0.003
Grade	< 0.001		
I		Reference	
II		1.252 (1.198–1.308)	< 0.001
III		1.685 (1.609–1.765)	< 0.001
Positive Lymph nodes	< 0.001		
0		Reference	
1–3		1.465 (1.413–1.519)	< 0.001
> 3		3.090 (2.969–3.216)	< 0.001
ER Status	< 0.001		
Positive		Reference	
Negative		1.353 (1.255–1.458)	< 0.001
PR Status	< 0.001		
Positive		Reference	
Negative		1.198 (1.154–1.243)	< 0.001
Radiation	< 0.001		
Yes		Reference	
No		1.323 (1.285–1.362)	< 0.001

*Other including American Indian/AK Native, Asian/Pacific Islander.

Abbreviations: CI; confidence interval; ER, estrogen receptor; HR, hazard ratio; IDC, infiltrating ductal carcinoma; ILC, infiltrating lobular carcinoma; PR, progesterone receptor.

### BCSS and competing-risk analysis

At 5 and 10 years after diagnosis, the cumulative incidences of death resulting from breast cancer (CIDBC) of the training cohort were 0.053 and 0.112, respectively, while the cumulative incidences of death resulting from other causes were 0.036 and 0.090, respectively. Estimates of CIDBC and other causes by clinicopathological variables are presented in Table 3. Patients younger than 40 years at diagnosis had the highest CIDBC (0.082/0.178 for 5/10 years), while patients between 50 and 59 years old at diagnosis had the lowest CIDBC than other ages (0.045/0.097 for 5/10 years; *P* < 0.001). Black patients had the highest CIDBC (0.100/0.185 for 5/10 years), while white and “other” patients had similar lower CIDBC (white, 0.050/0.107 for 5/10 years; “other”, 0.048/0.105 for 5/10 years; *P* < 0.001). There was no significant difference between different lateralities. Additionally, patients with ILC, histologic grade I, negative lymph node or positive ER/PR status had lower CIDBC, and patients with IDC, histologic grade III, more than 3 positive lymph nodes or negative ER/PR status had higher CIDBC (*P*'s < 0.001 for all). Receiving radiation decreased CIDBC from 0.064/0.128 for 5/10 years to 0.045/0.100 for 5/10 years (*P* < 0.001). All variables significantly correlated with CIDBC were used to build the nomogram to predict 5- and 10-year BCSS.

**Table 3:** Five- and 10-year cumulative incidences of death among patients in the training cohort

Variable	Cumulative Incidence of Death Resulting From Breast Cancer			Cumulative Incidence of Death Resulting From Other Causes		
	5-y	10-y	*P*	5-y	10-y	*P*
All Patients	0.053	0.112		0.036	0.090	
Age at diagnosis (y)			< 0.001			< 0.001
< 40	0.082	0.178		0.017	0.043	
40–49	0.049	0.113		0.009	0.021	
50–59	0.045	0.097		0.016	0.037	
60–69	0.048	0.099		0.039	0.098	
70–79	0.062	0.117		0.101	0.268	
Race			< 0.001			< 0.001
White	0.050	0.107		0.036	0.095	
Black	0.100	0.185		0.053	0.118	
Other	0.048	0.105		0.025	0.060	
Laterality			0.216			0.389
Left	0.054	0.113		0.037	0.093	
Right	0.052	0.111		0.036	0.095	
Tumor Size (cm)			< 0.001			0.006
≤ 2	0.024	0.060		0.033	0.091	
2–5	0.099	0.201		0.044	0.102	
> 5	0.198	0.339		0.047	0.094	
Histology type			< 0.001			0.012
IDC	0.055	0.113		0.036	0.093	
ILC	0.046	0.120		0.038	0.100	
Mix/Other	0.049	0.104		0.036	0.095	
Grade			< 0.001			< 0.001
I	0.012	0.033		0.034	0.095	
II	0.039	0.095		0.037	0.097	
III	0.113	0.206		0.038	0.088	
Positive Lymph nodes			< 0.001			< 0.001
0	0.022	0.054		0.035	0.097	
1–3	0.066	0.147		0.037	0.087	
> 3	0.205	0.373		0.044	0.089	
ER Status			< 0.001			< 0.001
Positive	0.051	0.110		0.036	0.095	
Negative	0.142	0.200		0.035	0.073	
PR Status			< 0.001			< 0.001
Positive	0.047	0.104		0.035	0.092	
Negative	0.085	0.157		0.042	0.103	
Radiation			< 0.001			< 0.001
Yes	0.045	0.100		0.025	0.073	
No	0.064	0.128		0.051	0.121	

Abbreviations: ER, estrogen receptor; IDC, infiltrating ductal carcinoma; ILC, infiltrating lobular carcinoma; PR, progesterone receptor.

### Nomogram

**Figure 2:**
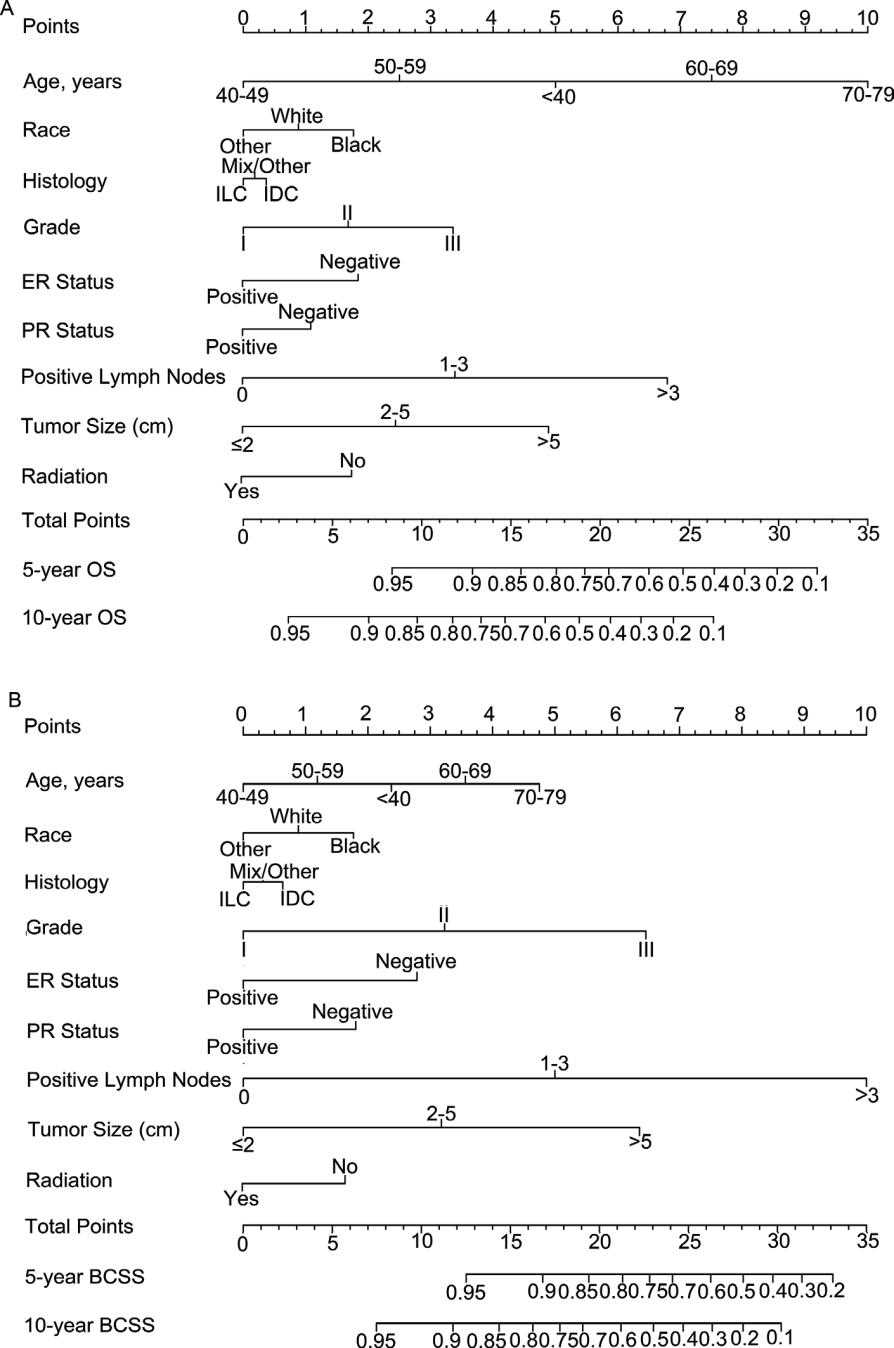
Nomogram for predicting 5- and 10-year (A) overall survival (OS) and (B) breast cancer-specific survival (BCSS) of luminal breast cancer patients Instructions for use of the nomogram: First, assign the points of each characteristic of the patient by drawing a vertical line from that variable to the points scale. Then, sum all the points and draw a vertical line from the total points scale to the 5- and 10-year OS or BCSS to obtain the probability of death. Abbreviations: ER, estrogen receptor; PR, progesterone receptor.

**Figure 3:**
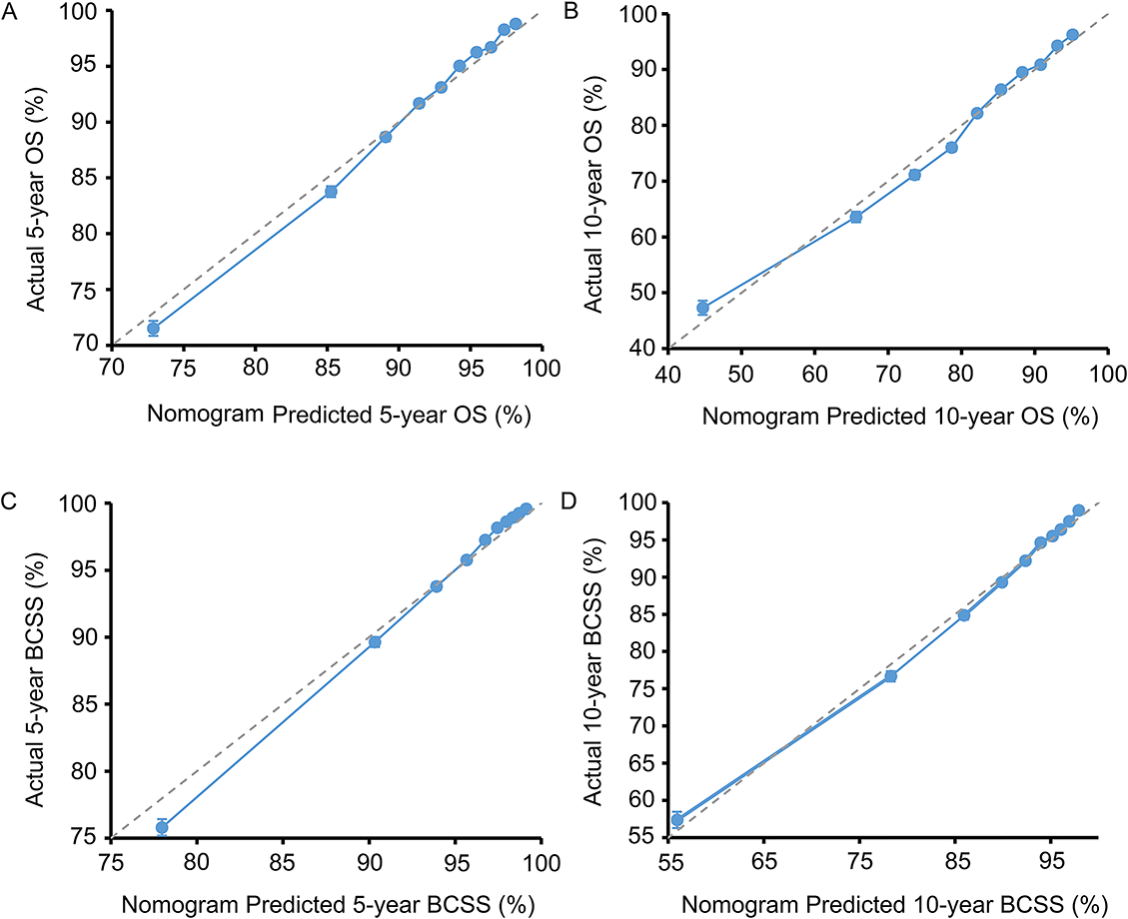
External calibration plot (**A**) 5-year and (**B**) 10-year overall survival (OS) nomogram calibration curves; (**C**) 5-year and (**D**) 10-year breast cancer-specific survival (BCSS) nomogram calibration curves. The dashed line represents a perfect match between the nomogram-predicted probability (*x*-axis) and the actual probability calculated by Kaplan-Meier analysis (*y*-axis). The cohort was divided into ten equal groups in sample size according to predicted probability of OS and BCSS. Closer distances from the points to the dashed line indicate better agreement between the predicted and actual outcomes.

Nomograms for predicting 5- and 10 year OS and BCSS were constructed based on the reduced multivariate models in the training cohort (Figure 2). Validation of the nomogram was processed both internally and externally. As shown in Table S1, in the internal validation cohort (training cohort), the Harrell's C-indexes for the nomograms to predict OS and BCSS were 0.732 (95% CI, 0.728 to 0.736) and 0.800 (95% CI, 0.795 to 0.804), respectively. In the external validation cohort, the C-indexes were slightly lower: 0.731 (95% CI, 0.727 to 0.735) and 0.794 (95% CI, 0.789–0.798), respectively. This finding implied that these models were reasonably accurate. The internal and external calibration plots of the CIF are presented in Figure S1 and Figure 3, revealing an excellent correlation in OS and BCSS between the nomogram and observed outcome.

To clarify applications of the nomograms, we can take two normal breast cancer patients for examples. Both of them were with IDC, ER positive, PR positive, 3 positive lymph nodes and didn't receive radiation after surgery. The first patients is an Chinese, aged 45 with grade I, 1 cm tumor while the other is black, 70 years old with grade III, 6 cm tumor. According to the nomograms, the Chinese patient gets 5.75 and 6 points in OS and BCSS nomograms, respectively, which meant that the patient's possibilities of 5-year OS, 5-year BCSS and 10-year BCSS are greater than 0.95 while 10-year OS is between 0.90–0.95. In the black patient condition, scores for OS and BCSS nomograms were 22.75 and 25.25, respectively. Thus for this patient, both 5-year OS and BCSS were less than 0.7, and 10-year OS and BCSS were less than 0.4. Based on the different hazard, we could treat these patients accordingly.

## DISCUSSION

In light of the heterogeneity and the high risk of late recurrence of luminal breast cancer, a brief nomogram based on long-term follow-up of a population-based cohort that predicts long-term OS and BCSS should be quite useful and practical for clinicians. To date, there is no comprehensive nomogram for luminal breast cancer based on a competing risks model, although several nomograms have been reported to predict prognosis of breast cancer in other subtypes. [11, 12] Using the SEER database with a mean follow-up of 107.4 months, we developed novel nomograms predicting the 5- and 10-year OS and BCSS of luminal breast cancer based on competing risks analysis. With these nomograms, clinicians can classify patients into different risk groups, thus making individualized treatment possible.

The log-rank test and Cox proportional hazards regression were used for calculating the independent prognostic factors of OS. However, these analyses cannot be used to identify prognostic factors of BCSS due to the possibility of leading to biased results. [13] Therefore, a competing risks model was introduced. A competing risk was defined when the occurrence of an event either precludes the occurrence of another event under evaluation or altered the probability of occurrence for the other event. [14] Due to the relatively good prognosis of some tumors, competing causes of mortality is a critical consideration when estimating the probability of death. Death from other causes was treated as a competing risk event in this study because it precludes the possibility of death resulting from breast cancer. [15] As shown in this study, luminal breast cancer patients with radical mastectomy registered in the SEER program from 1990 to 2006 had excellent prognosis. Five- and 10-year probabilities of death were 0.089 and 0.202, respectively. In addition, five-year CIDBC was 0.053. Interestingly, the ten-year CIDBC increased 0.059 to 0.112. This result indicated that more patients died due to breast cancer during the second five-year period. These results are consistent with former reports that more relapse events occurred 5 years after initial treatment than in the first 5 years. [6–9]

Several clinicopathological characteristics were proven to be independent prognostic factors for both OS and BCSS in the present study, including age at diagnosis, race, tumor size, histology type, grade, lymph node status, ER/PR status and radiation, which are in line with previous studies [16–21]. Age at diagnosis has been examined as an important prognostic factor in several studies. [22–24] In the nomograms, the hazard ratios of OS and BCSS in different age groups formed a U-shaped curve, with younger and older patients experiencing the worst survival while patients aged 40 to 59 years had the best survival in HoR positive breast cancer. These results were consistent with our recent report about the association of age and BCSM. [25] Although this phenomenon has attracted attention, the mechanism is still unclear. Previous data have described that black women have a high risk of breast cancer recurrence [26] and probability of death [27] and that black ethnicity is an independent predictor of poor survival [28]. In this study, white women have a better OS and BCSS than black women but relatively poorer OS and BCSS than Asian and “other” women.

Despite these strengths, there are limitations in our study. First, information regarding adjuvant therapy such as chemotherapy, endocrine therapy and targeted therapy is not available in the SEER database. Without the above information, the nomograms might result in bias when used to predict individual outcome even though several pivotal factors had been considered. Second, immunohistochemical markers including human epidermal growth factor 2 (HER-2) and Ki-67 were not recorded in the SEER database between 1990 and 2006, which might also impair the effectiveness of the nomograms. Furthermore, due to the retrospective nature of our study, these nomograms must be further validated in a prospective cohort or a clinical trial before being applied to clinical use.

In summary, we developed nomograms to estimate the probability of OS and BCSS of luminal breast cancer based on a large, population-based cohort with long-term follow-up. The nomograms we developed have perfect performance in both the training and validation cohorts, and they are potentially effective tools for predicting the prognosis of luminal breast cancer patients. Our nomograms will help clinicians identify individuals who are at high risk of overall mortality or BCSM within 5 or 10 years, thus providing more individualized treatment strategies.

## PATIENTS AND METHODS

### Patient screening and data processing

Data were obtained from the SEER program of the National Cancer Institute, which consists of 18 population-based cancer registries [29]. The inclusion criteria we used to identify eligible patients were as follows: female; aged 18 to 79 years old at diagnosis; known time of diagnosis between January 1, 1990 and December 31, 2006; diagnosis with unilateral breast cancer; diagnosis with breast cancer as the first and only cancer diagnosis; diagnosis confirmed in a living patient and not obtained from a death certificate or autopsy; surgical treatment with either mastectomy or breast-conserving surgery; pathological confirmation of invasive carcinoma; AJCC stage I-III; histological grade I-III; and known estrogen receptor (ER) and progesterone receptor (PR) statuses. Patients diagnosed before 1990 were not included because ER and PR status were not recorded in the SEER database until 1990. Additionally, to ensure adequate follow-up time, patients diagnosed after 2006 were not included. Patients with inflammatory breast cancer or Paget's disease and lack of data on any of the above inclusion criteria were also excluded. A total of 176,082 patients were included after the screening. Mixed infiltrating ductal carcinoma (IDC) and infiltrating lobular carcinoma (ILC) composition and other histological types were classified into “mix/other”. American Indian/Alaskan Native and Asian/Pacific Islanders were recorded as “other” under race. Continuous variables, such as age, number of positive lymph nodes and tumor size, were transformed into categorical variables based on recognized cutoff values.

### Statistical analysis and construction of the nomogram

To establish and validate a competing risks nomogram, the eligible patients were randomly divided into a training (*n* = 87,867) cohort and a validation (*n* = 88,215) cohort.

The median follow-up was estimated as the median observed survival time. OS was measured as the time from diagnosis to death, date of last follow-up or December 31, 2011 (if date of last contact was after 2011). Kaplan-Meier plots and log-rank tests were applied to determine univariate prognostic factors. A multivariate Cox proportional hazards model was applied to estimate the independent effects of the univariate prognostic factors on OS. The independent prognostic factors determined by the multivariate analysis were used to construct the nomogram for OS.

BCSS was measured as the time from diagnosis to death attributed to breast cancer, date of last follow-up or December 31, 2011 (if the date of last follow-up was after 2011). Deaths from other causes were considered to be a competing risk. The cumulative incidence function (CIF) was used to assess the probability of breast cancer-specific mortality (BCSM) and death from other causes. CIF was calculated by Gray's test between category groups. [30] A sub-distribution analysis of competing risks was performed to construct the competing risks model. [31] In the Cox regression model analyzing the cause-specific regression, patients who died from other causes were excluded at the end of follow-up. Integrating the associated risk factors, nomograms were developed to predict the risk of death and BCSM 5 or 10 years after diagnosis. All *P* values are two-sided, and those less than 0.01 were considered statistically significant on the basis of the large number of patients.

### Validation and calibration of the nomogram

To decrease overfit bias, the nomograms were subjected to 200 bootstrap resamples for internal validation in the training cohort and external validation in the validation cohort, respectively. The marginal estimate versus model average predictive probability was used to create a calibration diagram. The predictions should fall on a 45-degree diagonal line in a perfectly calibrated model. An index of probability of concordance (C-index) between predicted probability and actual outcome was calculated to evaluate the predicting ability and discrimination of the model. [32] The value of the C-index ranges from 0.5 to 1.0, with 0.5 indicating random chance and 1.0 indicating a perfectly corrected discrimination.

Identification of independent prognostic factors was conducted using SPSS 18.0 (SPSS, Chicago, IL, USA). Construction, validation and calibration of the nomogram were performed on R version 3.1.2 software (Institute for Statistics and Mathematics, Vienna, Austria; www.r-project.org). [33] The R packages cmprsk [34] and rms [35] and a C-index function for competing risks model [36] were used for modeling and developing the nomograms.

### Ethics statement

Our study was approved by Shanghai Cancer Center Ethical Committee. For cancer is a reportable disease in every state in the U.S, informed patient consent are not required for the data released by the SEER database.

## SUPPLEMENTARY FIGURE AND TABLE



## Abbreviations

ER: estrogen receptor
PR: progesterone receptor
SEER: Surveillance Epidemiology and End Results Database
OS: overall survival
BCSS: breast cancer specific survival
CIF: cumulative incidence function
BCSM: breast cancer-specific mortality
HoR: hormone receptor
ATAC: the Arimidex Tamoxifen Alone or in Combination
BIG: the Breast International Group
IDC: infiltrating ductal carcinoma
ILC: infiltrating lobular carcinoma
CIDBC: the cumulative incidences of death resulting from breast cancer
HER-2: human epidermal growth factor 2
CI: confidence interval.
