# Supplementary material for: Nomograms to estimate long-term overall survival and breast cancer-specific survival of patients with luminal breast cancer
Source: Oncotarget. 2016 Mar 7;7(15):20496–506. doi: 10.18632/oncotarget.7975 (PMC4991470; doi:10.18632/oncotarget.7975)
Supplement: Supplementary file 1 [file oncotarget-07-20496-s001.pdf]

Nomograms to estimate long-term overall survival and breast cancer-specific survival of patients with luminal breast cancer

Supplementary Materials

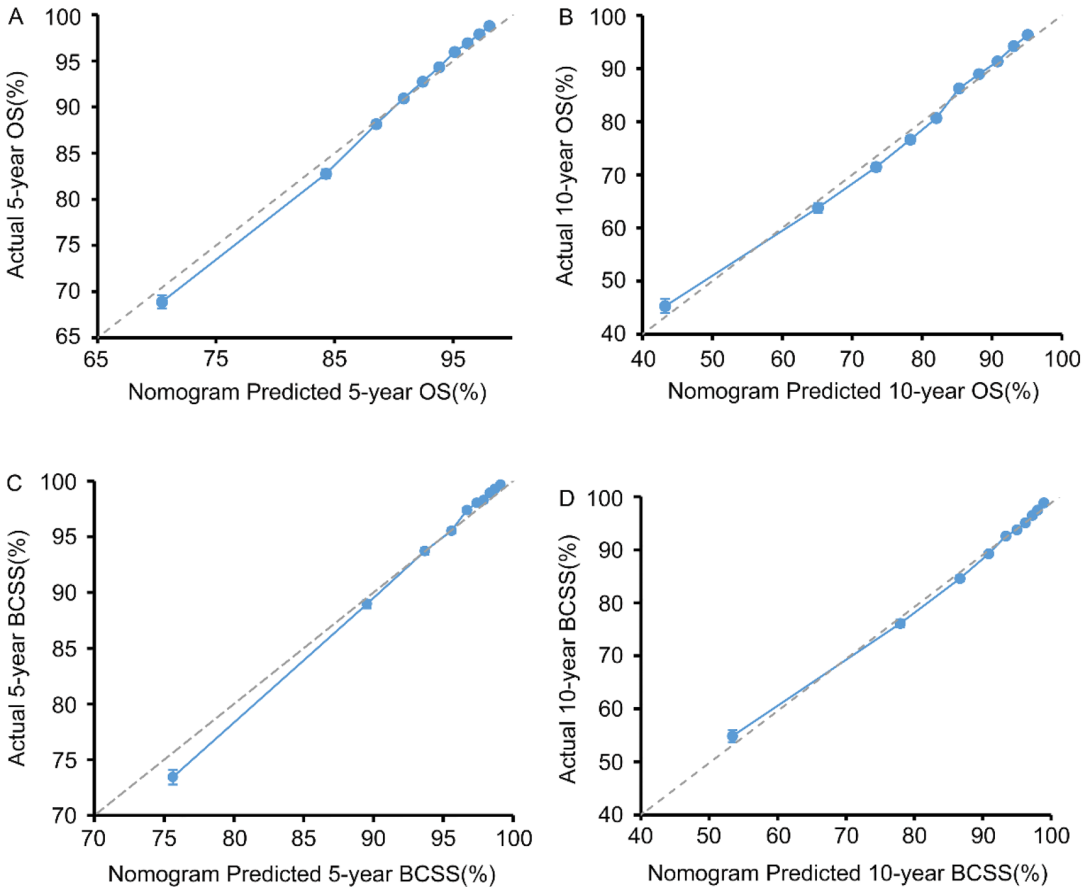

Supplementary Figure S1: Internal calibration plot. (**A**) 5-year and (**B**) 10-year overall survival (OS) nomogram calibration curves; (**C**) 5-year and (**D**) 10-year breast cancer-specific survival (BCSS) nomogram calibration curves. The dashed line represents a perfect match between nomogram-predicted probability (*x*-axis) and actual probability calculated by Kaplan-Meier analysis (*y*-axis). The cohort was divided into ten equal groups according to predicted probability of OS and BCSS. Closer distances from the points to the dashed line indicate better agreement between the predicted and actual outcomes.

Supplementary Table S1: The harrell's C-index for the nomogram to predict OS and BCSS

Groups	OS		BCSS	
	HR	95% CI	HR	95% CI
Training cohort	0.732	0.728–0.736	0.800	0.795–0.804
Validation cohort	0.731	0.727–0.735	0.794	0.789–0.798

Abbreviations: BCSS, breast cancer-specific survival; CI, confidence interval; HR, hazard ratio; OS, overall survival.
